# Differential roles of individual bile acid in physiology and disease

**DOI:** 10.1016/j.phrs.2025.107845

**Published:** 2025-06-29

**Authors:** Mingjie Fan, Zhiyu Yang, Lihua Jin, Wendong Huang

**Affiliations:** a School of Life Sciences, Shandong First Medical University & Shandong Academy of Medical Sciences, Jinan 250117, China; b Department of Endocrinology, Shandong Provincial Hospital Affiliated to Shandong First Medical University, Jinan, Shandong 250021, China; c Key Laboratory of Endocrine Glucose & Lipids Metabolism and Brain Aging, Ministry of Education, Shandong Provincial Hospital Affiliated to Shandong First Medical University, Jinan, Shandong 250021, China; d Department of Diabetes Complications and Metabolism, Arthur Riggs Diabetes & Metabolism Research Institute, Beckman Research Institute, City of Hope, Duarte, CA 91010, USA

**Keywords:** Bile acids, Cytochrome P450 family 8 subfamily B, member 1 (CYP8B1), 12α-hydroxylated BAs, Bile acid receptor, Gut microbiota, Diseases

## Abstract

Bile acids (BAs) have originally been linked to nutrient digestion and absorption, however, emerging research underscores their pivotal role as signaling molecules in regulating multiple physiological and pathological processes. Alternations in BA levels and profiles are frequently observed in a variety of diseases, indicating their potential as either diagnostic markers or therapeutic targets or agents. In addition to the levels of BAs, the specific composition of BA species, such as primary vs. secondary BAs, conjugated vs. unconjugated BAs, and hydroxylated vs. non-hydroxylated BAs, plays a critical role in maintaining physiological balance and modulating disease pathogenesis. In this review, we highlight the association between 12alpha-hydroxylated (12α-OH) BAs and non-12α-OH BAs, as well as other BA modifications, with diverse diseases, including liver diseases, gastrointestinal conditions, metabolic syndromes, age-related neurological diseases, and cancers. While substantial progress has been made in elucidating the pleotropic role of BAs in disease mechanisms, the clinical translation of BAs as diagnostic markers or therapeutic targets or agents requires further validation and standardization. Ongoing discoveries in this dynamic field are paving the way for breakthroughs in both mechanistic insight and clinical translation.

## 1. Introduction

**Fig. 1.**
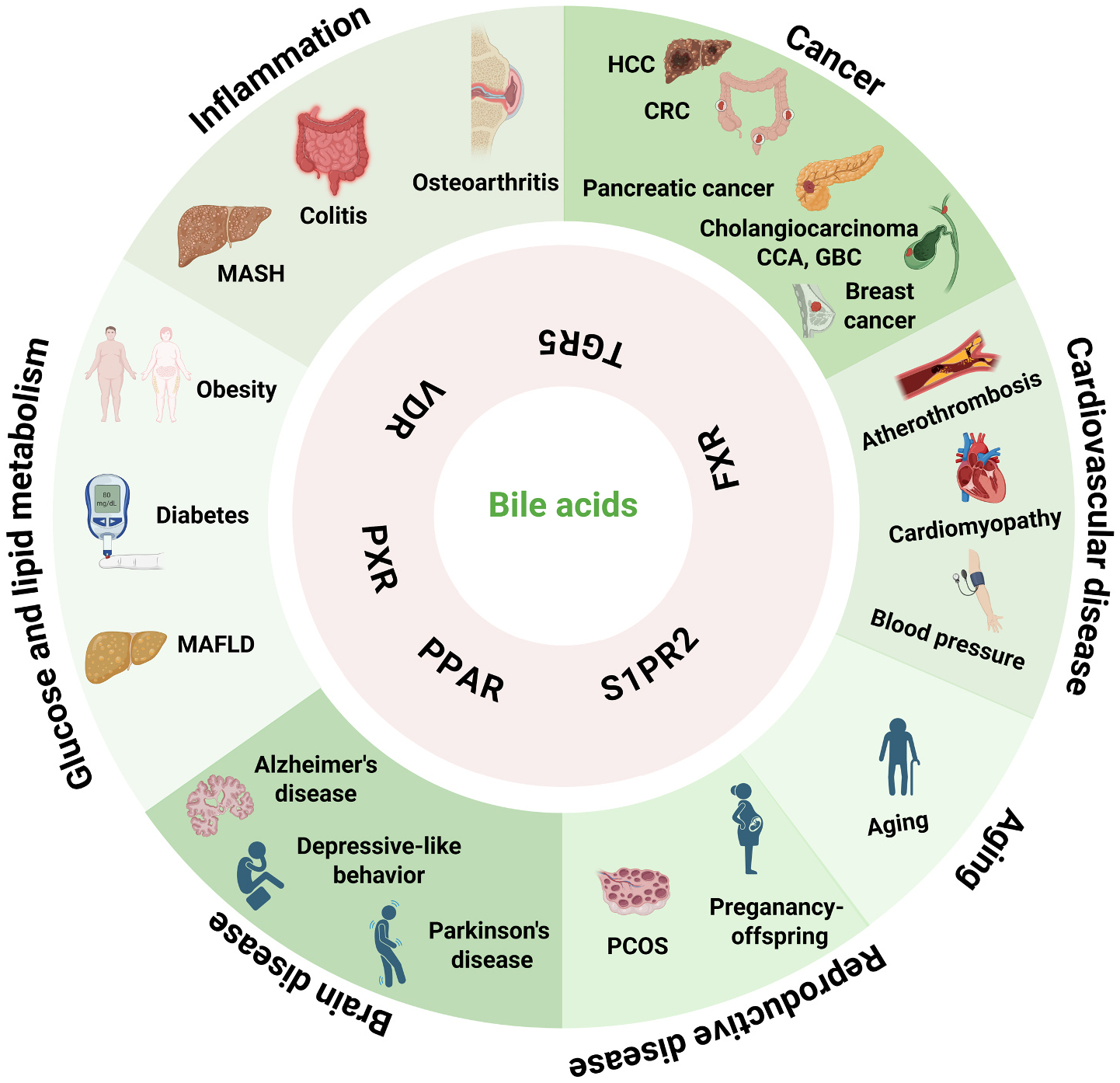
Involvement of bile acids and their receptors in diverse diseases. BAs exert pleiotropic effects by activating or inhibiting their receptors, including FXR, TGR5, PPAR, VDR, PXR, and S1PR2. These pathways modulate diverse diseases, such as metabolic disorders (e.g., obesity, diabetes, MAFLD), inflammatory conditions (e.g., MASH, colitis, osteoarthritis), and cancers (e.g., HCC, CRC, CCA, GBC, pancreatic cancer, and breast cancer). BAs also contribute to cardiovascular diseases (e.g., atherothrombosis, cardiomyopathy, hypertension) and aging-related neurodegenerative disorders (e.g., Alzheimer’s disease, Parkinson’s disease, depressive-like behavior). Emerging evidence further implicates BAs in reproductive system pathologies (e.g., PCOS and pregnancy). **Abbreviations:** FXR, farnesoid X receptor; TGR5, Takeda G protein-coupled receptor 5; PPAR, peroxisome proliferator-activated receptor; VDR, vitamin D receptor; PXR, pregnane X receptor; S1PR2, sphingosine-1-phosphate receptor 2; MAFLD, metabolic dysfunction- associated fatty liver disease; MASH, metabolic dysfunction-associated steatohepatitis; HCC, hepatocellular carcinoma; CRC, colorectal cancer; CCA, cholangiocarcinoma; GBC, gallbladder cancer.

Bile acids (BAs) are amphipathic molecules that facilitate dietary lipids absorption or excretion in the small intestine [1]. BAs also serve as hormones regulating multiple signaling pathways in physiological conditions [2]. BAs primarily mediate their biological effects through interactions with two key receptors: the nuclear farnesoid X receptor (FXR) [3] and membrane-bound Takeda G protein-coupled receptor 5 (TGR5) [4–6]. By modulating these receptor pathways, BAs play crucial regulatory roles in diverse physiological processes and disease states, including cholesterol homeostasis [7,8], glucose metabolism [9–11], lipid regulation [12], as well as inflammation [13,14]. BAs also regulate physiological functions via other receptors [15], such as nuclear receptors vitamin D receptor (VDR) [16], pregnane X receptor (PXR) [17], peroxisome proliferator-activated receptor (PPAR) [18] and sphingosine 1-phosphate receptor 2 (S1PR2) [19]. Dysregulation of BA homeostasis causes various metabolic diseases, such as obesity [20], diabetes, non-alcoholic fatty liver (NAFLD) [21] and alcoholic-associated liver disease (AALD) [22], 23]. In addition, BAs have been linked to cardiovascular disease (CVD) and heart failure [24,25], and inflammatory immune signaling in the gastrointestinal tract such as inflammatory bowel disease (IBD) [26], colitis [27], pancreatitis [28], aging [29], neurodegenerative disease [30,31] and cancers such as hepatocellular carcinoma (HCC) [32] and colon cancer [33]. Recently, it was reported BAs also play a critical role in polycystic ovary syndrome by regulating the human gut microbiome and also participate in some hereditary reproductive disorders [34,35] (Fig. 1).

Although the total BAs or BAs profile could be a stratification of NAFLD [36,37], there are no specific BAs that are identified as reliable biomarkers for health/disease indicators. Due to the presence of multiple types of circulatory BAs in the digestive tract and portal vein, the functions of specific BAs and their potential as diagnostic markers and therapeutic targets for diseases have been underappreciated. While the collective functions of bile acids are well-documented, recent advances highlight that individual bile acids—depending on their hydroxylation patterns, conjugation states, and receptor selectivity—exert distinct, sometimes opposing effects[38,39]. Although the topic of the specific BAs and their interaction targets is vast, recent reviews have yet to cover the functions of specific BAs comprehensively.

Conventional classification of bile acids is generally based on either conjugation status or the primary/secondary categorization, where secondary bile acids are defined by microbial transformation. In this review, we primarily focus on specific BAs, particularly non-12α-hydroxylated (non-12α-OH) BAs and 12α-hydroxylated (12α-OH) BAs, which are controlled by Cytochrome P450 family 8 subfamily B member 1 (CYP8B1, also known as sterol 12-alpha-hydroxylase). In addition, we classify the specific BAs in relation to the intestinal microbiome and their corresponding signaling pathways to provide an overview of recent research and highlight the potential to combine BAs and microflora treatment and diagnosis for diseases.

## 2. CYP8B1 as a key enzyme in controlling the 12α-OH BA to non-12α-OH BA ratio

### 2.1. CYP8B1 catalyzes 12α-OH BAs biosynthesis

**Fig. 2.**
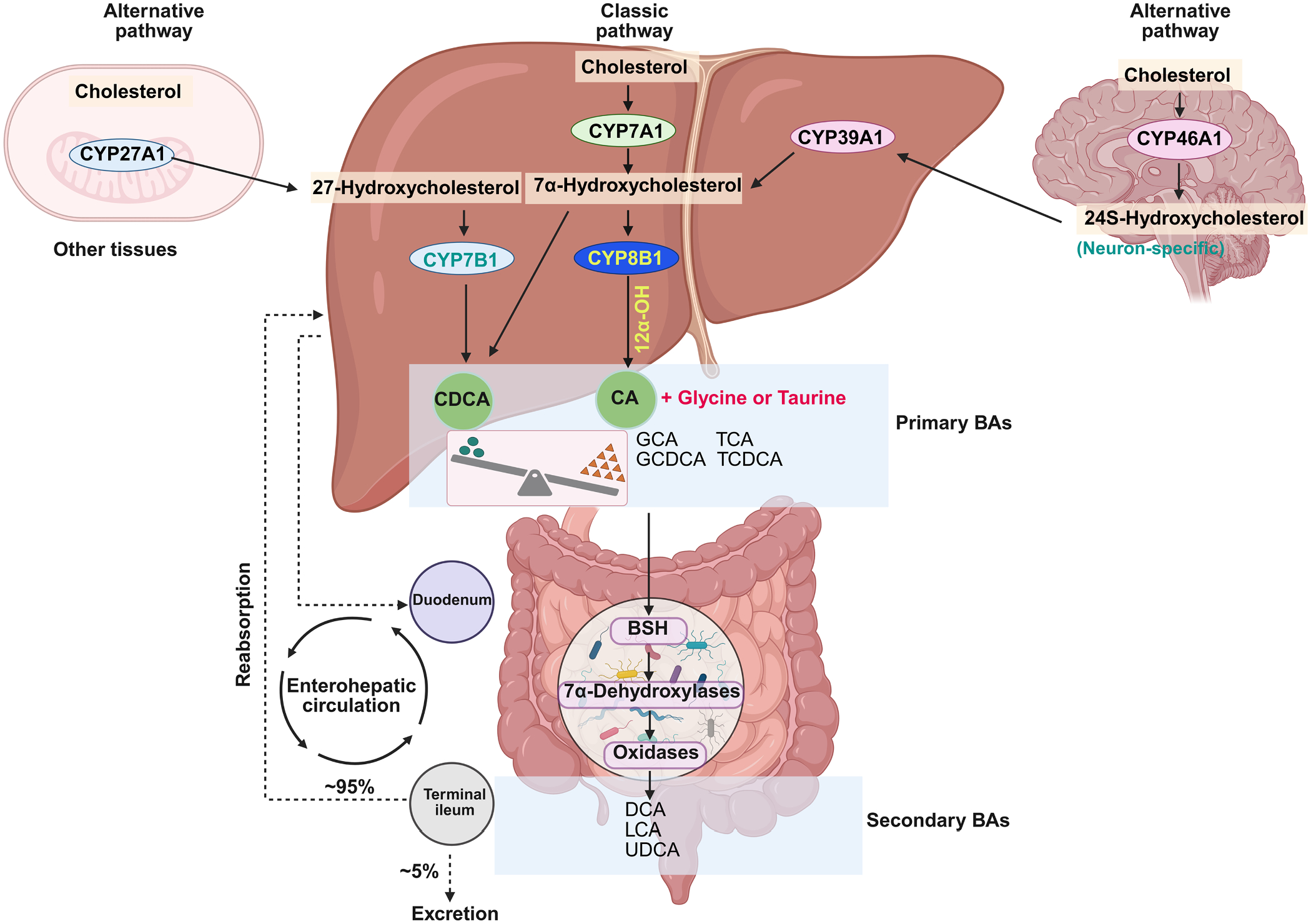
Bile acid biosynthesis and enterohepatic circulation. Bile acids (BAs) are synthesized via two major pathways: [1] The classic pathway initiated by CYP7A1 in hepatocytes, where cholesterol undergoes an enzymatic cascade culminating in CA production via CYP8B1-mediated 12α-hydroxylation; and [2] The alternative pathway originating from mitochondrial CYP27A1, with CYP7B1 catalyzing the formation of CDCA of other tissues. In the brain, cholesterol is first converted to 24S-hydroxycholesterol by CYP46A1, then transported to hepatocytes for conversion to CA via CYP39A1. Newly synthesized BAs are primarily conjugated with glycine or taurine before storage in the gallbladder. Gut microbiota metabolizes primary BAs into secondary bile acids, including LCA, DCA, and UDCA. Most BAs are reabsorbed in the ileum via passive diffusion or active transport to return through portal vein back to hepatocytes, completing the enterohepatic circulation (~95 %). A small fraction is excreted in feces and urine (~5 %). **Abbreviations:** CYP7A1, cholesterol 7α-hydroxylase；CYP8B1, sterol 12α-hydroxylase; CYP27A1, sterol 27-hydroxylase; CYP7B1, oxysterol 7α- hydroxylase; CYP39A1, CYP46A1, 24-hydroxylase; CYP39A1, oxysterol 7α-hydroxylase; CA, cholic acid; CDCA, chenodeoxycholic acid; LCA, lithocholic acid; DCA, deoxycholic acid; UDCA, ursodeoxycholic acid; BSH, bile salt hydrolase.

BAs are initially synthesized from cholesterol with a tightly regulated cascade, primarily orchestrated by two pathways: the classic pathway in the liver initiated by cholesterol 7α-hydroxylase (CYP7A1), and the acidic pathway in other tissues or human infant which initiated by mitochondrial sterol 27-hydroxylase (CYP27A1) [40]. Cytochrome P450 enzyme 46A1 (CYP46A1), predominantly expressed in the brain, catalyzes the formation of 24S-hydroxycholesterol from cholesterol in neurons [41]. Subsequently, these intermediates are transformed into chenodeoxycholic acid (CDCA) in humans and α/β muricholic acid (α/βMCA) in mice [42]. These, along with cholic acid (CA), constitute the primary bile acids. In humans, primary bile acids are predominantly conjugated with glycine, while in mice, they are conjugated with taurine. Upon secretion into the intestinal tract, primary bile acids undergo crucial deconjugation and transformation into secondary bile acids by microbiota flora possessing bile salt hydrolases (BSHs) activity [43,44]. Approximately 95 % of bile acids reaching the distal ileum are reabsorbed into the portal vein and return to the liver [45,46] (Fig. 2). This tightly coordinated synthesis, modification, and enterohepatic recirculation of BAs underscores their essential role in maintaining systemic metabolic homeostasis.

### 2.2. CYP8B1-driven 12α-OH BA production regulate the metabolic process via BA receptors

**Fig. 3.**
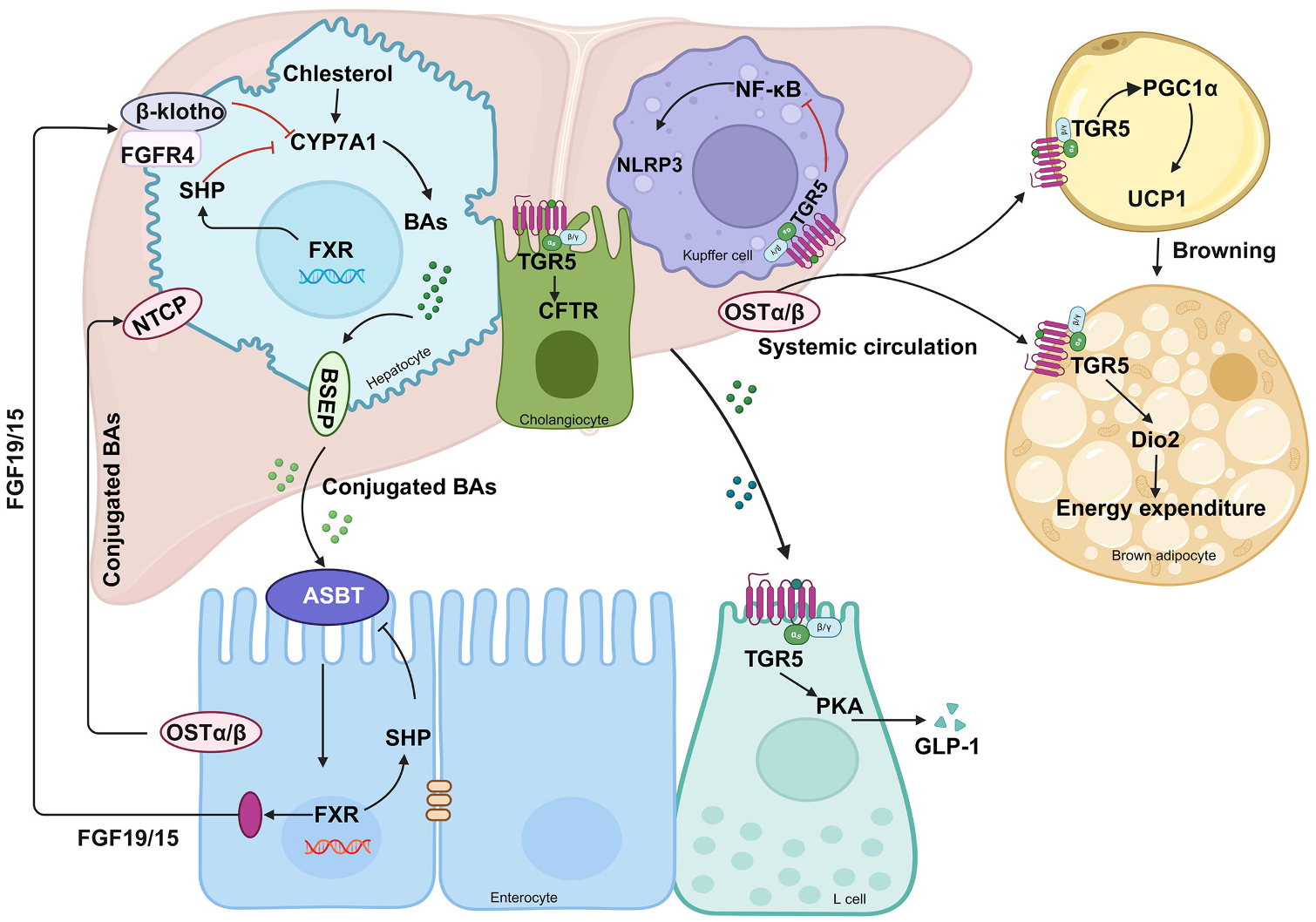
Bile Acid-Mediated Signaling Through FXR and TGR5 Receptor Pathways. In the liver, cholesterol is converted to primary BAs via the CYP7A1. These BAs activate the nuclear receptor FXR, FXR activation directly induces SHP (small heterodimer partner) to downregulate CYP7A1. Subsequntely, BAs secret into intestine induces enterocyte FGF15/19 secretion and inverse to suppress hepatic CYP7A1 through the FGFR4/β-Klotho receptor, forming a negative feedback loop. In TGR5 mediated regulation: [1] BAs inhibit NF-κB-mediated inflammation in Kupffer cells through TGR5 while promoting HCO_3_^−^ secretion via FXR-CFTR in cholangiocytes; [2] BA-activated TGR5 stimulates GLP-1 secretion from intestinal L cells; and [3] In adipose tissue, BAs enhance energy expenditure by activating the DIO2/T3 thermogenic pathway in brown fat and inducing UCP1-mediated browning of white fat. Solid arrows indicate activation.

CYP8B1 is exclusively expressed in the liver, where it catalyzes the conversion of 7α-hydroxy-4-cholesten-3-one into 7α,12α-dihydroxy-4-cholesten-3-one[47]. 12α-OH BAs, including CA and DCA, are structurally defined by a hydroxyl group at the C-12α position of the steroid nucleus, a key feature that differentiates them from non-12α-OH subclasses such as CDCA and LCA (Table 1) [48]. CYP8B1 is the only enzyme to produce 12α-OH BAs, thereby acting as a switch to control the ratio of 12α-OH BAs to non-12α-OH BAs in the BA pool [49]. Here, we place emphasize the significance of CYP8B1 in modulating the ratio of 12α-OH BAs to non-12α-OH BAs. Studies demonstrated that *Cyp8b1*^*−/−*^ mice increased synthesis of CDCA, resulting in elevated energy expenditure, which help protect against obesity [50]. Inhibition of CYP8B1 activity enhances skeletal insulin resistance in humans by increasing the CDCA level [51]. Direct regulation of *Cyp8b1* expression by hepatic growth hormone can alters the BA profile. Disruption of the growth hormone receptor signaling induces insulin resistance and gut microbiota dysbiosis [52]. We also identified CYP8B1 as a downstream target benefiting from vertical sleeve gastrectomy (VSG) in mice [53]. Importantly, both BA subclasses predominantly exert their biological functions through activation of BA receptors - particularly the two major receptors as mentioned FXR and TGR5 (Fig. 3).

As a nuclear receptor, FXR is abundantly expressed throughout human tissues, most prominently in the liver and intestine, where it functions as a primary sensor and regulator of BA homeostasis[54]. Among endogenous ligands, the non-12α-OH CDCA is the most potent natural FXR agonist, followed by its conjugated forms (TCDCA/GCDCA) and other BAs with varying affinities[55]. Notably, the efficacy of BA-mediated FXR activation depends on structural features which influenced the hydrophobicity of BA as ordered by LCA>DCA>CD-CA>CA>HDCA>UDCA> β-MCA> ω-MCA>their glycine conjugates>their taurine conjugates [3,56]. Upon activation in the liver, FXR initiates a negative feedback loop that suppresses bile acid synthesis [57]. FXR activation directly induces SHP (small heterodimer partner), which inhibits the transcription factors LRH-1(liver receptor homolog-1) and HNF4α (hepatocyte nuclear factor 4α) to downregulate CYP7A1 in the classical bile acid synthesis pathway [58–60]. Additionally, FXR stimulates intestinal secretion of FGF19/15, which subsequently signals through hepatic FGFR4/β-Klotho receptors to further suppress CYP7A1 expression [61–63]. It is well established that CYP8B1 expression is regulated by liver FXR. Administration of 3,5,3’-triiodo-L-thyronine (T3) suppresses CYP8B1 expression in hypothyroid mice, leading to increased non-12α-OH that are FXR antagonistic BAs. This, in turn potentiates GLP-1 production and insulin secretion by repressing intestinal FXR signaling [64,65].

While FXR serves as the master regulator of BA homeostasis through nuclear signaling, the membrane receptor TGR5 mediates complementary metabolic effects—particularly through 12α-OH BA-dependent activation with LCA and its taurine conjugate (TLCA) serving as the most efficacious agonists (E_C_50 ~0.3–1 μM), followed by DCA and its conjugated forms, while weaker activation is observed with CDCA and its conjugates [66,67]. Through cAMP-dependent signaling, TGR5 activation exerts pleiotropic metabolic effects, including the induction of thermogenesis in brown adipose tissue via type 2 deiodinase (DIO2)-mediated T3 production and promotion of white adipose tissue browning through PGC-1α/UCP1 upregulation [22,68,69]. In the intestine, TGR5 stimulates GLP-1 secretion from L-cells to enhance glucose homeostasis, while in the liver it modulates glycogen synthesis and inhibits inflammatory responses through suppression of NF-κB and NLRP3 inflammasome activity in macrophages [70,71]. Additionally, TGR5 activation in cholangiocytes promotes cytoprotective bicarbonate secretion via cystic fibrosis transmembrane conductance regulator (CFTR) to mitigate cholestatic injury [72,73]. These multifaceted roles position TGR5 as a promising therapeutic target for metabolic disorders including diabetes, obesity, NAFLD/NASH, and atherosclerosis, leveraging its ability to simultaneously improve glycemic control, enhance energy expenditure, and reduce tissue inflammation. These findings regarding CYP8B1 functions suggests that it is a potential therapeutic target for metabolic diseases based on its role in controlling the 12α-OH BA to non-12α-OH BA ratio. Thus, CYP8B1-driven 12α-OH BA production not only shapes FXR activity but also calibrates TGR5 signaling amplitude, offering a single enzymatic node to coordinately regulate BA receptor networks

## 3. Role of non-12α-OH BAs in physiology and diseases

### 3.1. Non-12α-OH BAs as modulators of lipid and glucose metabolism

**Fig. 4.**
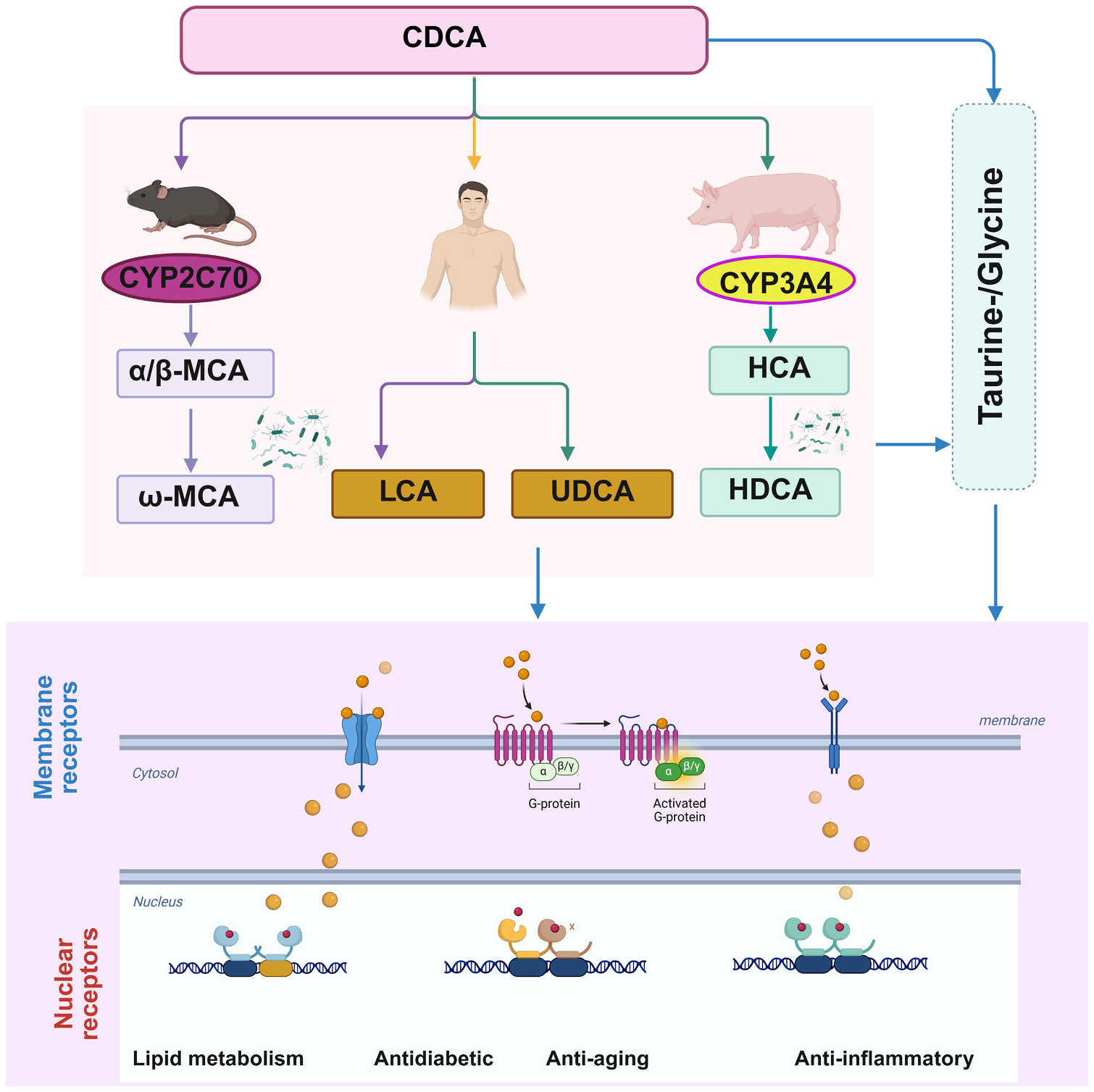
Metabolic regulatory functions of non-12α-hydroxylated BAs across different species. CDCA, the primary non-12α-hydroxylated bile acid (BA), shows distinct metabolic fates across different species: [1] In mice, CDCA is converted to α/βMCA via CYP2C70, followed by transformation to the secondary bile acid ωMCA. [2] In humans, CDCA remains the primary form and is metabolized by gut microbiota to LCA or epimerized to UDCA. [3] In pigs, CDCA is hydroxylated by CYP3A4 to HCA, with further microbial conversion to HDCA. These primary or secondary BAs, either in free form or conjugated with taurine/glycine, function as signaling molecules in diverse diseases progression by activating the membrane G protein-coupled bile acid receptor (TGR5), or entering the nucleus to modulate nuclear receptor activity. **Abbreviations:** CDCA, chenodeoxycholic acid; α/βMCA, α/β-muricholic acid; ωMCA, ω-muricholic acid; LCA, lithocholic acid; UDCA, ursodeoxycholic acid; HCA, hyocholic acid; HDCA, hyodeoxycholic acid; TGR5 - Takeda G protein-coupled receptor 5; CYP2C70, cytochrome P450 2C70; CYP3A4, cytochrome P450 3A4.

Non-12α-OH BAs encompass primary BAs CDCA, α/βMCA and their taurine (T) or glycine (G) conjugated forms T/G-CDCA, T/G-α/βMCA, as well as the secondary BAs including LCA, UDCA, HDCA, and their conjugated forms. In mice, MDCA, ω-MCA and HDCA act as non-12α-OH secondary BAs derived from α/βMCA, respectively. In humans, they originate from CDCA via gut microbiome conversion [15]. Additionally, hyocholic acid (HCA), also known as gamma-muricholic acid (γMCA) in mice, is synthesized from CDCA by the enzyme Cytochrome P450 Family 3 Subfamily A Member 4 (CYP3A4) in pig [74]. Understanding the generation and modulation of these non-12α-OH BAs may open new avenues for targeting metabolic or inflammatory diseases (Fig. 4).

Fecal HCA decreases in prediabetes people but rises in serum of post-bariatric surgery patients, suggesting its metabolic benefits. ωMCA and HCA could increase the GLP1 secretion, which subsequently exhibit antidiabetic effects by stimulating TGR5 activity in mice [75]. Under the transformation of microbiome, HCA turn into HDCA, and HDCA alleviates NAFLD by simultaneously activating hepatic CYP7B1 and PPARα which further enhances lipid metabolism [76,77]. Dietary supplementation of HDCA ameliorates diet-induced NAFLD in male mice by activating fatty acid oxidation in a hepatic PPARα-dependent manner [18].

Additionally, UDCA attenuated high-fat diet induced obesity by enhancing the levels of non-12-OH BAs [78]. Lower level of isoursodeoxycholate (iso-UDCA) may contribute to increased satiety and improved lipid control after bariatric surgery [79]. Treatment with FXR agonists, CDCA and obeticholic acid (OCA), significantly reduces lipid peroxidation by upregulating the ferroptosis-inhibitory regulators, providing potential benefits against ferroptosis-mediated degenerative diseases [80]. Pu-erh tea reduces intestinal microbiome BSH activity, resulting in increased level of conjugated BAs such as TCDCA and TUDCA in mice, and GCDCA and GUDCA in human. This suggests a potential therapy for hypercholesterolemia and hyperlipidemia [81]. Hydrophobic BAs generally act as FXR agonists. Additionally, hydrophilic non-12α-OH BAs such as TMCA, UDCA, and T/GUDCA function as endogenous FXR antagonists [82]. Melatonin reduces deconjugation of TUDCA to inhibit aging-triggered high-level expression of hepatic FXR to ameliorate hepatic lipid dysmetabolism [83].

### 3.2. Non-12α-OH BAs as dual regulators of inflammation

TCDCA and GCDCA have been shown to induce TGR5-dependent anti-inflammatory responses following thrombocytopenia syndrome virus infection [86]. The anti-inflammatory function of non-12α-OH BAs has been demonstrated in studies involving coronavirus [88]. UDCA as a secondary BAs in human, mediated downregulation of angiotensin converting enzyme 2 by suppressing FXR signaling has been found to reduce susceptibility to SARS-CoV-2 infection in vitro and in vivo, as well as in human lungs and livers perfused ex-situ [89]. A novel bile-based assay, BileMet, identified the CDCA glycine conjugate as a protective metabolite associated with biliary tract cancer in three independent cohort [84]. Its secondary form UDCA exerts cytoprotective effects in hepatocytes through inhibiting FXR activity in the regulation of chronic hepatitis [90,91]. It has been demonstrated that UDCA alleviates liver fibrosis in bile duct ligation mice and enhances liver regeneration in partial hepatectomy mice [98]. UDCA reverses the impact of TCA on immune evasion and liver tumor growth in HCC patients [99,87]. UDCA is widely used for treating patients with primary biliary cirrhosis (PBC) and a variety of chronic cholestatic liver diseases [100]. LCA is a secondary bile acid produced by gut microbial metabolism of CDCA. High level of TLCA also showed protective effects via inhibition of ferroptosis after virus infection [93]. iso-LCA most potently and dose-dependently suppressed NK cell secretion of IFN-γ and TNF-α, leading to HCC development [32]. Microbiota-derived 3-oxo-Δ4,6-LCA inhibit bladder cancer tumor growth by androgen receptor[94]. It can recapitulate the effects of calorie restriction in mice and activation of AMP-activated protein kinase (AMPK) inducing anti-aging effects [95]. TβMCA was markedly elevated in aging mice and accumulated in brain resulting in aging-related neuro-impairment [101].

In the context of neurological diseases, variations in the BA profile have been observed. CDCA and UDCA were identified as distinct BA signatures for patients with Parkinson’s disease (PD) with mild cognitive impairment (PD-MCI) [41,85]. Increased GUDCA significantly protects dopaminergic neurons against rotenone-induced damage of PD patients [102]. The study demonstrated that incorporating BA features (CA, CA %, DCA/CA, and GDCA/CA) significantly enhanced the predictive performance of clinical markers for MCI progression in male patients, with DCA/CA and GDCA/CA ratios progressively increased with advancing MCI severity [103]. Exercise upregulated taurine and UDCA level mediating the protective effects on bone mass alleviates osteopenia [104]. Furthermore, GUDCA level was significantly reduced in osteoarthritis patients, supplementation of GUDCA inhibits FXR activity and mitigates osteoarthritis progression in mice [92]. The results were consistent with another study that administration of GUDCA has been shown therapeutic effects on atherosclerosis by reducing the levels of circulating ceramides and cholesterol through inhibition of the FXR signaling [105].

### 3.3. Non-12α-OH BAs as therapeutic agents: from natural compounds to synthetic derivatives in metabolic diseases

Considering the functions of non-12-OH BAs, particularly UDCA, HDCA and their derivates, they are primarily regarded as drugs or biomarkers, either alone or in combination with first-line drugs for various diseases. Our team initially identified notoginsenoside Ft1 from *Panax notoginseng* as an agonist of TGR5. Ft1 treatment elevated serum free and taurine-conjugated BAs, including LCA, TLCA, TCDCA and α/βMCA, all of which are non-12α-OH BAs thereby alleviating high-fat diet induced obesity and insulin resistance in mice [21]. Another study identified higher levels of hepatic primary BAs, including β-MCA, omega-MCA (ω-MCA), and TCDCA, after treatment with saikosaponins component. These elevated levels may account for the hepatoprotective and cholagogic effects of *Radix Bupleuri* in mice [106]. Quercetin treatment substantially promotes the generation of non-12OH BAs, particularly UDCA and LCA, which further activates TGR5-dependent thermogenesis in brown adipose tissue and induced browning of white adipose tissue [107]. BAR501, an alcohol derivative of UDCA with potent and selective TGR5 activity, has been shown to reverse insulin resistance, ameliorate liver histology. It can promote the browning of epididymal white adipose tissue and increase energy expenditure in a rodent model of NASH [96]. The acidic pathway intermediate 7α-hydroxyl-3-oxo-4-cholestenoic acid (7-HOCA) accumulates intracellularly in CRAT-deficient cardiomyocytes, provoking mitochondrial DNA stress that activates the cGAS-STING pathway and subsequent type I interferon responses [97]. Some BAs combine with PD-1 show significant anti-tumor effects [99]. In summary, specific non-12α-OH BAs participate in improving metabolic illness phenotype by interacting with FXR/TGR5, offering significant potential for further clinical therapy study.

## 4. Role of 12α-OH BAs in physiology and disease

### 4.1. Metabolic and Hepatic Impacts of 12α-OH BAs

**Fig. 5.**
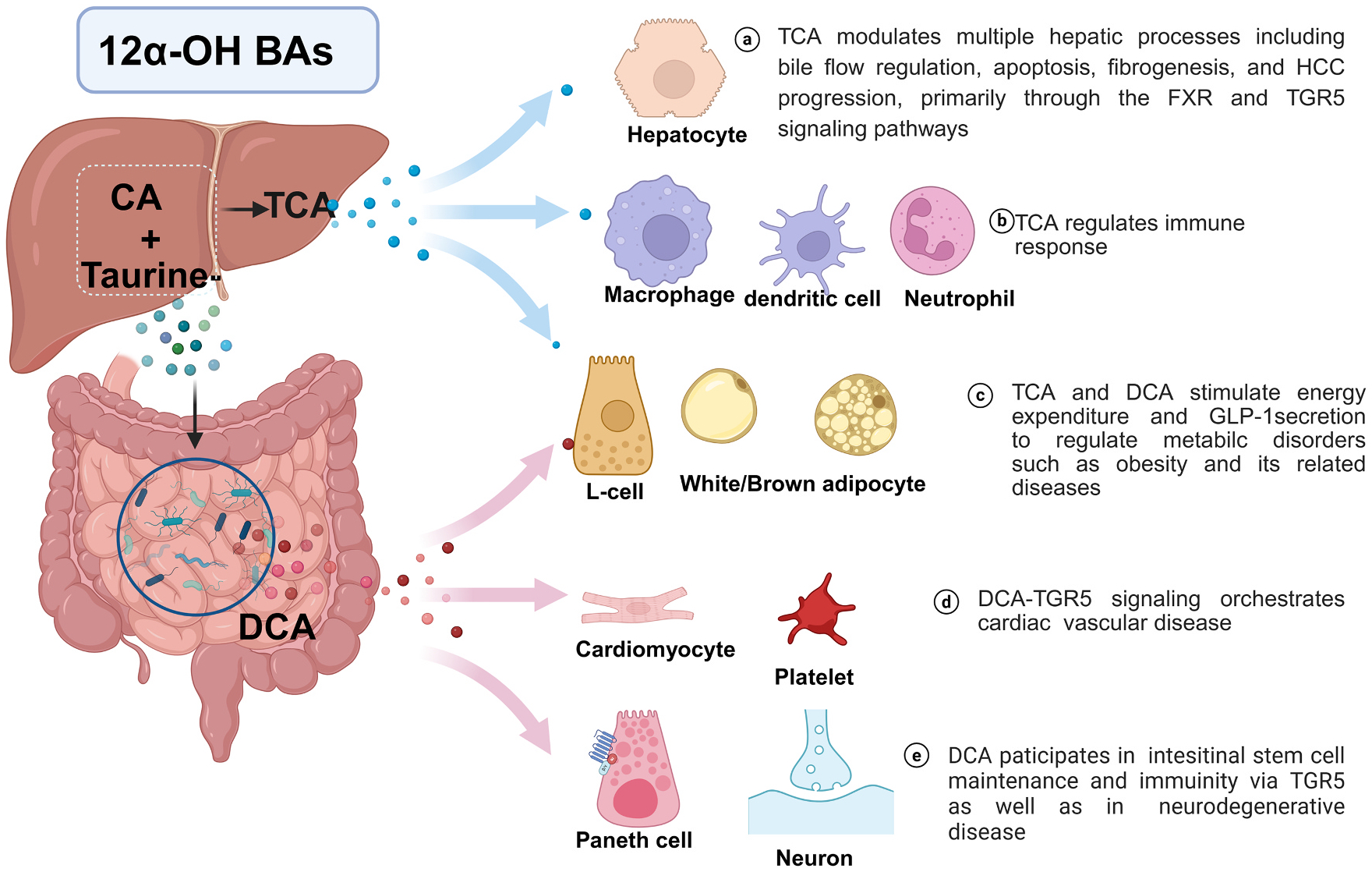
12α-OH BAs mediate pathological effects through receptor signaling in target cells. The major 12α-OH BAs include CA, T/GCA, and DCA. These BAs function as endogenous agonists for TGR5 and modulate pathological processes in target cells. Key cellular targets and pathological effects: [1] Metabolic regulation: TCA/DCA modulate lipid and glucose metabolism in hepatocytes, brown/white adipocytes, and intestinal L-cells; [2] Immune modulation: 12α-OH BAs influence inflammatory responses through immune cells (macrophages, dendritic cells, neutrophils) in inflammation-related diseases; [3] Cardiovascular effects: 12α-OH BAs regulate cardiac and vascular function via cardiomyocytes and platelet TGR5 signaling pathways; [4] Neurological and epithelial effects: DCA participates in neurodegenerative diseases and maintains intestinal stem cells through Paneth cell regulation. **Abbreviations:**12α-OH BAs, 12α-hydroxylated bile acids; CA, cholic acid; T/GCA, taurocholic acid/glycocholic acid; DCA, deoxycholic acid; TCA, taurocholic acid; TGR5, Takeda G protein-coupled receptor 5; FXR, farnesoid X receptor.

12α-OH BAs, including CA, T/GCA, and the secondary form DCA exert critical roles in hepatic and metabolic regulation [108,109] (Fig. 5). Insulin mitigates hypercholesterolemia by suppressing CYP8B1-mediated 12α-hydroxylated bile acid (12α-OH BA) production, thereby reducing intestinal cholesterol absorption [110]. The CYP8B1-CA axis suppresses PPARα-mediated fatty acid oxidation in intestinal stem cell (ISC) *Lgr5*^+^, impairing ISCs renewal and exacerbating colitis, as evidenced by elevated CA and CYP8B1 activity in IBD patients and mice [111]. TCA promotes HBV replication by inhibiting CD8^+^ T and natural killer cells function in chronic hepatitis B patients [112] and are positively correlated with increased M2-like tumor-associated macrophages in HCC [113]. Elevated TCA also drives immune evasion in HCC, reversible by modulating the 12α-OH/non-12α-OH BA ratio [90]. Additionally, TCA and GCA stimulate inflammatory response by phosphorylating JAK and STAT3 in murine models of cholestasis [114]. Furthermore, FXR deficiency exacerbates TCA accumulation in iron-induced hepatotoxicity in rodents [115]. These findings highlight the dual role of 12α-OH BAs in both aggravating and protecting against hepatic disorders.

### 4.2. Systemic and microbiome-derived effects of DCA

The secondary 12α-OH BA- DCA emerges as a key modulator of systemic physiology. Generated from TCA via microbial deconjugation in the intestine and converted to DCA [116,117]. DCA level was significantly upregulated after alcohol consumption, activating TGR5-mediated brown fat energy expenditure in alcoholic-associated liver disease mice model [22]. However, DCA disrupts intestinal homeostasis by inhibiting the Wnt signaling pathway in Paneth cell, affecting the maintenance of antimicrobial immunity during disease [118]. Paradoxically, DCA exhibits organ-specific benefits: it improves cardiac function after myocardial infarction via TGR5/FXR pathways [67,119,120] and suppresses agonist-induced platelet activation and thrombus formation through interaction with platelet TGR5 receptors [24]. In the gut, TDCA corelated with the number of infiltrating CD4^+^ and CD8^+^ T cells contributed to the enhanced ileitis through TGR5 signaling [5]. While, in the brain, accumulated DCA impairs cognition by activation of TGR5 in both wild-type mice and neurons derived from AD patients [121]. Conjugated 12a-OH BAs, such as TDCA and GDCA, significantly increased in NASH patients and liver fibrosis mouse models via TGR5 mediated p38MAPK and ERK1/2 signaling [122]. Collectively, DCA play pivotal roles in disease pathogenesis by functioning as endogenous agonists of TGR5

### 4.3. Mechanistic insights and therapeutic opportunities

The 12α-OH/non-12α-OH BA ratio is a pivotal determinant of disease outcomes. Dietary TCA supplementation blunts the beneficial effects of VSG [123]. In comparison to DSS-induced colitis, untreated *Mdr2*^−*/*−^ mice showed an increased TCA signal in an inflammatory bowel disease-primary sclerosing cholangitis (IBD-PSC) mouse model, leading to a protective response against cholestatic liver disease triggered by colitis [124]. This suggests potential for multi-organ treatment strategies for PSC. Iron metabolism is primarily regulated by the liver, and ferroptosis represents an iron-dependent form of non-apoptotic cell death [125]. Ferroptosis is implicated in liver [126,127], brain [128], kidney [129,130], and heart pathology [131]. Increased FXR expression promotes intestinal epithelial cell ferroptosis in necrotizing enterocolitis (NEC), suggesting that targeting intestinal FXR and ferroptosis pathways may have therapeutic potential for NEC treatment [132]. Taken together, 12α-OH BAs function as endogenous regulators of FXR, TGR5, and immune pathways, offering targets for metabolic, hepatic, and neurodegenerative disorders.

## 5. Role of BA – gut microbiota interplay in metabolic disease regulation

### 5.1. Bidirectional regulation between gut microbiota and bile acid metabolism

The gut microbiota has garnered significant attention as a remarkable regulator involved in host diseases and health status [61,133]. Increasing research suggests that gut microbiota dysbiosis has a significant adverse effect on the host [134]. Microbes metabolize BAs, and in turn, BAs influence the composition and diversity of the gut microbiota regardless non-12α-OH BAs or 12α-OH BAs. Alterations in the composition of the gut-associated microbial community are associated with many human illnesses [117,135]. A study profiling of gut microbiota and BAs under physiological conditions revealed inverse correlations: GCA with *Anaerostipes hadrus* and *Faecalibacterium prausnitzii*; TCA/TCDCA ratio with *Alistipes putredinis* and *Bilophila wadsworthia*; and TDCA with *A. putredinis* [136]. *Clostridium difficile* infection is a major public health concern, with TCA promoting spore formation while CDCA inhibits its growth [43,137]. These findings collectively underscore the gut microbiota-BA axis as a tightly coupled system whose disruption contributes to disease, offering actionable targets for microbiome-based therapies.

### 5.2. Therapeutic potential of microbial BA modulation

*Parabacteroides distasonis* (*P. distasonis*) is a key commensal bacterium with multifaceted roles in human health, influencing metabolic and inflammatory conditions including diabetes [138], colorectal cancer [139], arthritis [140] and obesity [141]. Therapeutic administration of *P. distasonis* demonstrates significant metabolic modulation: it reduces TCDCA levels to suppress hepatocyte pyroptosis and alleviate hepatic fibrosis [142] while elevating beneficial LCA and UDCA concentrations along with gut succinate levels[141]. Notably, this bacterium exhibits a bidirectional relationship with BAs - its abundance is increased by HDCA [76,77] but diminished during calorie restriction, leading to reduced non-12α-OH UDCA/LCA levels and potentially contributing to post-diet weight rebound [143]. These findings collectively position *P. distasonis* as a promising probiotic candidate for metabolic and hepatic disorders.

*Akkermansia muciniphila (A. muciniphila)* demonstrates multifaceted therapeutic potential through BA modulation. Its secreted metabolite harmaline specifically upregulates hepatic bile acid-CoA: Amino Acid N-Acyltransferase (BAAT) enzyme expression, thereby enhancing the synthesis of TCDCA [86]. *A. muciniphila* -induced the fat mass and obesity-associated protein upregulation enhances CYP8B1 expression, driving cholesterol-to-CA conversion. CA subsequently activates adipose FXR to suppress lipid accumulation [144]. In MAFLD-MASH progression, *A. muciniphila* plays a protective role: while disease is associated with elevated DCA/LCA levels, the depletion of 3-sulfated cholic acid (3-sucCA) inversely correlates with severity. Mechanistically, 3-sucCA from probiotic Bacteroides uniformis enriches *A. muciniphila* populations, reinforcing intestinal barrier function [91] Reciprocally, UDCA treatment promotes *A. muciniphila* colonization, ameliorating DSS-induced colitis [145]. Beyond metabolic effects, *A. muciniphila* - derived BA metabolites improve neuropsychiatric outcomes by rescuing CRS-induced 5-HT dysfunction and depressive-like behaviors [146].

Individuals with polycystic ovary syndrome showed markedly elevated *Bacteroides vulgatus*(*B. vulgatus*)*, which is* accompanied by reduced GDCA and TUDCA levels in serum [34]. Elevated TCA and DCA negatively correlated with *B. vulgatus* and *Ruminococcus torques*, suggesting a potential for alleviating obesity [147]. However, another study reported that GUDCA positively regulates TLCA levels and *B. vulgatus* abundance, which further activates TGR5 and UCP1 signaling to play anti-T2DM effects [148]. Patients with CVD exhibited significantly reduced serum DCA levels, concomitant with decreased abundance of the *B. vulgatus*, which is implicated in DCA metabolism [24]. These context-dependent effects highlight how *B. vulgatus* functions as a microbial rheostat, with its metabolic impact determined by specific BA interactions and disease microenvironments.

Osteoarthritis patients showed reduced gut *Clostridium bolteae* (*C. bolteae*) levels, positively correlating with serum GUDCA [92]. Transplantation of *Clostridium sp*. and supplementation of UDCA could ameliorate statin-induced glucose intolerance in statin induced hyperglycemia [149]. Administration of *Clostridium scindens* in mice increased the levels of hepatic non-12-OH BAs [78,150]. 3-O-acylation-CA, a novel type of acylated secondary BAs depleted in T2DM is generated from the gut commensal *Christensenella minuta*, targeting enterohepatic signaling axis to alleviate abnormal glucose and lipid metabolism in host [151]. Anti-α4β7-integrin treatment in humanized colitis mice elevated CDCA and LCA levels, concurrent with enrichment of *Paraprevotella, Prevotella, Roseburia, Lactobacillus, and Clostridium*, enhancing gut barrier function and mitigating intestinal inflammation [152–154]. Li et.al elucidated a positive correlation between the gut microbial genus *Eubacterium_R* and HDCA, which could explain the lower plasma HDCA level in sepsis patients with reduced abundance of *Eubacterium_R* [155]. Colonization with *Turicibacter* strains H121 led to an increase in GβMCA; however, strains MOL361 and 1E2 decreased taurine-conjugated primary BAs [156]. Our previous work confirmed that a reduced *Firmicutes*-to-*Bacteroidetes* ratio correlates with increased tauromuricholic acid (TMCA) levels and decreased obesity after VSG [70]. Recently, another study further revealed that this microbial ratio modulates BA profile to suppress FXR signaling, thereby mitigating chlorothalonil-induced obesity in mice [157]. The *Ruminococcaceae* family serves as a specific link to dominating the generation of LCA and DCA [158]. DCA and GCDCA aggravated *B. fragilis*-induced cholestasis by inhibiting FXR signaling and promoting hepatic BA accumulation, thereby contributing to intrahepatic cholestasis of pregnancy (ICP) [159].

In colorectal cancerous tissues, altered mucosal microbiota with increased levels of the genera *Bacteroides*, *Curtobacterium*, and *Campylobacter*, along with an increase in DCA, has been observed [160]. This finding could pave the way for the development of potential therapeutic targets for colorectal cancer. TDCA promotes normal gastric epithelial cell proliferation with LPS-producing *Prevotella melaninogenica*, suggesting a critical role in gastric carcinogenesis [161]. In contrast, *Parabacteroides goldsteinii* maintains gut homeostasis through associations with beneficial bile acids (Ta/β-MCA, UDCA, 7-keto-LCA) - a relationship disrupted by aspirin treatment, leading to impaired barrier function and stem cell dysregulation [162]. In summary, as the master modulators of bile acid metabolism, gut microbes orchestrate host physiology via metabolite-receptor networks, offering both disease biomarkers and druggable targets for microbiota-directed therapies.

## 6. Conclusions and perspectives

BAs have garnered increasing attention across various research fields. Despite their involvement in numerous physiological and pathological processes, BAs remain underutilized as diagnostic markers or therapeutic targets or agents largely due to the complexity and diversity of BA classifications. A deeper understanding of specific BA signaling pathways holds promise for identifying molecular targets relevant to different diseases, including those affecting the liver, intestines, heart, brain, cardiovascular system, and even cancer. In this review, we examined recent publications with a focus on CYP8B1 and specific BA 12α-OH modifications. We categorized them into non-12α-OH BAs and 12α-OH BAs in mice and humans. Non-12α-OH BAs, such as CDCA and α/β-MCA, along with their conjugated forms (T/G-CDCA, T/G-α/β-MCA), were found to be altered in various diseases and may serve as potential diagnostic markers. Additionally, both non-12α-OH and 12α-OH BAs can act as signaling molecules by activating or inhibiting receptors such as FXR and TGR5. The interaction between BAs and intestinal flora plays a crucial role in regulating many diseases, particularly those of the gastrointestinal system. However, the relationship between specific BAs and specific gut bacteria remains incompletely understood. Studies have shown that the microbiota can transform and modify different BAs, converting CA into DCA, and CDCA or α/β-MCA into LCA, UDCA, HDCA, HCA, etc. along with their conjugated forms. We have summarized and classified the relationship between BAs and microbiota, which may open new therapeutic avenues for BAs, gut microbiota, or their combination with first-line clinical drugs for disease treatment.

## Figures and Tables

**Table 1:** Structural comparison of major species-specific BAs and their hydroxylation patterns.

Abbreviation	BA name	Hydroxylations	Conjugates	Primary/Secondary
CA	Cholic acid	3α, 7α, 12α	GCA, TCA	Primary
DCA	Deoxycholic acid	3α, 12α	GDCA, TDCA	Secondary
CDCA	Chenodeoxycholic acid	3α, 7α,	GCDCA, TCDCA	Primary
LCA	Lithocholic acid	3α	GLCA, TLCA	Secondary
UDCA	Ursodeoxycholic acid	3α, 7β	GUDCA, TUDCA	Secondary*
				Primary^#^
^#^α-MCA	α-Muricholic acid	3α, 6β, 7α	Gα-MCA, Tα-MCA	Primary
^#^β-MCA	β- Muricholic acid	3α, 6β, 7β	Gβ-MCA, Tβ-MCA	Primary
^#^ω-MCA	ω- Muricholic acid	3α, 6α, 7β	Gω-MCA, Tω-MCA	Secondary
^#^MDCA	Murideoxycholic acid	3α, 6β	GMDCA, TMDCA	Secondary
^HCA	Hyocholic acid	3α, 6α, 7α	THCA, GHCA	Primary

# BAs in mice;

^ BAs in pig;

* UDCA in human is secondary BA

**Table 2:** different non-12a-OH BAs and their biological/pathological roles.

BA/Derivative	Biological/Pathological Role	Mechanism/Target	Associated Condition	Ref
Primary non–12α-OH BAs				
CDCA	- PD-MCI biomarker - Anti-ferroptosis	FXR agonism	PD-MCI, ferroptosis-related diseases	[80] [84] [41, 85]
TCDCA/GCDCA	- Anti-inflammatory - Hepatoprotective - Obesity improvement	TGR5 activation	Viral infections, biliary tract cancer, obesity	[81, 86]
α/βMCA	- improves metabolic health -Hepatoprotective	TGR5/FXR modulation	Obesity, liver injury	[21]
HCA (in pig)	- Metabolic benefits - GLP–1 secretion	TGR5 activation	Prediabetes, diabetes	[75]
Secondary non–12α-OH BAs				
UDCA	- Neuroprotective -Anti-inflammatory	FXR suppression,	PD-MCI, COVID–19, liver diseases, osteopenia	[79, 87] [88, 89] [90, 91]
T/GUDCA	- Neuroprotective (PD) -Osteoarthritis progression -Anti-atherosclerotic	FXR inhibition	PD, osteoarthritis, atherosclerosis	[82] [83, 92]
HDCA	- Alleviates NAFLD via fatty acid oxidation	CYP7B1/PPARα activation	NAFLD, obesity	[18, 76]
LCA/TLCA	- Anti-ferroptosis (post-viral) -improves obesity -improve insulin resistance	Ferroptosis inhibition, TGR5 activation	Viral infections, obesity	[93] [21]
ωMCA	- ↑ GLP–1 secretion - Hepatoprotective	TGR5 activation BA synthesis modulation	Diabetes liver injury	[75] [106]
Derivative BAs				
3-oxo-Δ4,6-LCA	- Anti-bladder cancer - Mimics calorie restriction (AMPK activation)	Androgen receptor, AMPK	Bladder cancer, aging	[94] [95]
BAR501	-Reverses insulin resistance -Promotes adipose browning - Ameliorates NASH	TGR5 agonism	NASH, obesity	[96]
7-HOCA	-Triggers cGAS-STING respon- ses in cardiomyocytes	Mitochondrial DNA stress	Cardiovascular disease	[97]

**Abbr.** 7-HOCA, 7α-hydroxyl-3-oxo-4-cholestenoic acid;

**Table 3:** Functional Classification of Gut Microbiota in BA Metabolism and Disease.

Microbial Taxon	Taxonomic Classification	Critical Enzymes	Metabolites Produced	Disease Associations	Therapeutic Potential	Ref.
*P. distasonis*	*Bacteroidetes*	7α/β-dehydroxylases	LCA, UDCA, 7-keto-LCA	Liver fibrosis, T2DM, obesity	Metabolic probiotic candidate	[138–142]
*A. muciniphila*	*Verrucomicrobia*	Sulfotransferases	3-sucCA, TCDCA	MAFLD, depression, colitis	FDA-approved probiotic	[86,91,144,146,163]
*B. vulgatus*	*Bacteroidetes*	BSH	TLCA, GDCA	T2DM (protective), PCOS (harmful)		[24,34,147,148]
*C. bolteae*	*Firmicutes (Clostridia)*	7α-dehydroxylase	LCA, UDCA	Intestinal inflammation, ICP	Pathobiont	[92]
*C. scindens*	*Firmicutes (Clostridia)*	7α-dehydroxylase	LCA, DCA	C. difficile infection, metabolic disorders	Live biotherapeutic product candidate	[78,150]
*Christensenella minuta*	*Firmicutes (Clostridia)*	Acyltransferase	3-O-acylation-CA	T2DM (depleted)	Phase I investi-ational probiotic	[139,151]
*Roseburia spp*.	*Firmicutes (Clostridia)*	BSH	Butyrate, unconjugated BAs	Obesity, colorectal cancer	Next-gen probiotic	[153]
*Lactobacillus spp*.	*Firmicutes (Bacilli)*	BSH	Unconjugated BAs	IBS, antibiotic-associated diarrhea	Traditional probiotic	[154]
*Eubacterium_R*	Firmicutes (Clostridia)	6α-hydroxylase	HDCA	Sepsis, energy metabolism disorders	Emerging therapeutic target	[155]
*Turicibacter*	*Firmicutes (Clostridia)*	Glycosyltransferases	GβMCA	Metabolic syndrome, obesity	Probiotic candidate	[156]
*Ruminococcus*	Firmicutes (Clostridia)	Undefined	DCA/TLCA	Obesity promotion, ICP	Potential therapeutic target	[159]
*Campylobacter spp*.	Proteobacteria	Undefined	Undefined	Colorectal cancer	Antibiotic target	[26,116,160]
*Ruminococcaceae family*	Firmicutes (Clostridia)	7α-dehydroxylase	LCA, DCA	Obesity, T2DM, Gastric disease, Cognitive	Microbial consortium target	[161,164]
*B. fragilis*	Bacteroidetes	Undefined	DCA, TCA	Colorectal cancer, ICP	Pathobiont	[159]
*P. goldsteinii*	Bacteroidetes	7α-HSDH	UDCA, 7-keto-LCA	Gut barrier dysfunction	Aspirin-alternative target	[162]

Abbr. 3-oxolithocholic acid, 3-oxoLCA; 3-sulfated cholic acid, 3-sucCA; 7-keto-lithocholic acid, 7-keto-LCA; Polycystic ovary syndrome, PCOS; Type 2 diabetes, T2DM; Metabolic-associated fatty liver disease, MAFLD; Metabolic-associated steatohepatitis, MASH; Intrahepatic cholestasis of pregnancy, ICP.
